# Designing and Testing an IoT Low-Cost PPP-RTK Augmented GNSS Location Device

**DOI:** 10.3390/s24020646

**Published:** 2024-01-19

**Authors:** Domenico Amalfitano, Matteo Cutugno, Umberto Robustelli, Giovanni Pugliano

**Affiliations:** 1 Department of Electrical Engineering and Information Technology, University of Naples Federico II, 80125 Naples, Italy; domenico.amalfitano@unina.it; 2 University of Benevento Giustino Fortunato, 82100 Benevento, Italy; 3 Department of Engineering, University of Naples Parthenope, 80143 Naples, Italy; umberto.robustelli@uniparthenope.it; 4 Department of Civil, Architectural and Environmental Engineering, University of Naples Federico II, 80125 Naples, Italy; giovanni.pugliano@unina.it

**Keywords:** PPP-RTK, IoT, GNSS, Point Perfect, high-accuracy positioning, low-cost hardware, mass-market navigation, u-blox ZED-F9P

## Abstract

Nowadays, the availability of affordable multi-constellation multi-frequency receivers has broadened access to accurate positioning. The abundance of satellite signals coupled with the implementation of ground- and satellite-based correction services has unlocked the potential for achieving real-time centimetre-level positioning with low-cost instrumentation. Most of the current and future applications cannot exploit well-consolidated satellite positioning techniques such as Network Real Time Kinematic (RTK) and Precise Point Positioning (PPP); the former is inapplicable for large user bases due to the necessity of a two-way communication link between the user and the NRTK service provider, while the latter necessitates long convergence times that are not in keeping with kinematic application. In this context, the hybrid PPP-RTK technique has emerged as a potential solution to meet the demand for real-time, low-cost, accurate, and precise positioning. This paper presents an Internet of Things (IoT) GNSS device developed with low-cost hardware; it leverages a commercial PPP-RTK correction service which delivers corrections via IP. The main target is to obtain both horizontal and vertical decimetre-level accuracies in urban kinematic tests, along with other requisites such as solution availability and the provision of connection ports for interfacing an IoT network. A vehicle-borne kinematic test has been conducted to evaluate the device performance. The results show that (i) the IoT device can deliver horizontal and vertical positioning solutions at decimetre-level accuracy with the targeted solution availability, and (ii) the provided IoT ports are feasible for gathering the position solutions over an internet connection.

## 1. Introduction

Today, everyone can harness signals delivered by the four Global Navigation Satellite Systems (GNSS) [1,2]. The advent of readily available and cost-effective multi-constellation multi-frequency GNSS receivers has significantly expanded access to accurate and precise positioning. Typically, in open-sky conditions, more than twenty GNSS satellites are visible above the horizon at any given time in any location. This abundance of satellite signals, along with the deployment of ground- and satellite-based data correction services, has triggered the potential to reach centimetre-level positioning accuracy in real time [3]. High-accuracy GNSS is not new, as surveyors and other professionals have had access to it for decades; nevertheless, high device costs and dependence on expensive correction services have prevented the technology from expanding outside of this niche market. Today, reaching centimetre-level positioning accuracy is achievable with affordable equipment thanks to the market availability of multi-constellation multi-frequency low-cost receivers offering Real Time Kinematic (RTK) capabilities. Additionally, commercial data correction services are continuously improving their coverage and performance, further enhancing the accuracy of cost-effective solutions. The plethora of present and future applications that can benefit from this technical advancement is awesomely extensive, including autonomous ground vehicles [4], urban micro-mobility [5], low-cost mobile mapping systems [6], unmanned aerial vehicles [7], smartphone positioning [8], and precision agriculture [9], among others. These mass market applications have all the common requirements mandatory for large-scale industrial production involving the employment of low-cost instrumentation. Well-consolidated techniques such as Precise Point Positioning (PPP) and Network RTK (NRTK) are inapplicable for kinematic applications with large user bases. This is mostly due to one factor; indeed, the limitations of NRTK stem from the need for a two-way communication link between the user and the NRTK service provider. This requirement arises because the service provider must generate corrections data based on the user’s position, and this computation must be performed individually for each user. As the number of users increases, the computational burden on service providers escalates, making them incapable of efficiently providing corrections to a substantial number of users, e.g., a massive number of potential autonomous driving users in the future. The correction data in NRTK are encoded and transmitted as an Observation Space Representation (OSR), where bandwidth constraints prevent a large number of users from accessing the service simultaneously. In contrast to NRTK, PPP and PPP-RTK techniques make use of State Space Representation (SSR), a concept introduced by [10]. SSR employs one-way communication, broadcasting a single stream of correction data to all rovers within a serviced area, which is a notable advantage over OSR. This approach represents and separates each error component, reducing the required bandwidth and enabling the resolution of phase ambiguity for single-frequency rovers [11]. SSR stands out as the current state of the art in high-precision GNSS positioning, meeting stringent requirements in bandwidth, flexibility, scalability, performance, and coverage that make it suitable for mass market needs. Although PPP reduces demands on communication infrastructure, its deployment in kinematic applications is limited due to extended convergence times. To overcome this limitation, the hybrid PPP-RTK technique has emerged as a potential solution to meet the demand for real-time, accurate, precise, and robust positioning in mass market kinematic applications. The fundamental concept underlying PPP-RTK involves enhancing the PPP technique by leveraging precise undifferenced atmospheric corrections and satellite clock corrections derived from a constellation of Continuously Operating Reference Stations (CORS). In contrast to the large-scale networks used in PPP, PPP-RTK utilises corrections from CORS within local networks, resulting in more accurate ionosphere and troposphere modelling. This approach represents the triggering factor that allows PPP-RTK to shorten convergence times. Starting from the pioneering work of [10], numerous researchers have devised various methods to accurately resolve undifferenced phase ambiguities and calculate an integer ambiguity fixed PPP solution, giving rise to the PPP-RTK technique. In general, PPP-RTK methods can vary in the models employed, the applied corrections, and the estimation strategies. In recent years, the research community has developed various processing models: ref. [12] proposed a multi-frequency (three) phase-only PPP-RTK model that excludes code observables and applied it to the Beidou constellation; additionally, ref. [13] proposed a PPP-RTK method with augmentation from a single reference station. Various researchers have integrated a PPP-RTK-capable GNSS receiver with an Inertial Navigation Unit (IMU) to provide accurate and reliable navigation along with bridging capabilities in GNSS-denied environments [14,15,16]. Among these, ref. [14] tested a PPP-RTK/INS TC in semi-urban and urban scenarios, finding solution availability with a horizontal error less than 0.2 m on 100% and 96.9%, respectively. The experiment reported in [15] reached solution availability as well, with a horizontal positioning error within 10 cm on 96.1% for multi-frequency PPP-RTK/INS with a fixing percentage of 90.9%. The findings of [16] showed accuracy of MEMS/PPP-RTK/INS/Vision of 6, 5, and 10 cm in the East, North, and Up components, respectively, with an ambiguity fixing rate of 83.6% in typical urban environments. Moreover, research works have reported the implementation of the PPP-RTK technique within smartphones [17,18]. In light of the central role of ionosphere modelling, particular attention has been paid to this aspect in the research community [19,20,21,22,23]. Wang and Zhang [24] developed a concept of all-frequency PPP-RTK tested in a vehicle-borne experiment carried out in Wuhan City; their results showed that the percentage of the epochs with a horizontal positioning error of less than 0.1 m was increased by 5–30% from conventional to all-frequency PPP-RTK. The performance of two low-cost receivers, namely, the u-blox zed-F9P mounted on a C099 board and the u-blox zed-F9R equipped with an inertial platform and mounted on C102 board, were investigated by [25]; their findings showed sub-decimetric accuracy in an urban area. A tightly integrated PPP-RTK/MEMS/vision model was developed by [26]. The results achieved in urban environments showed a sub-decimetric accuracy of 4.1, 2.2, and 7.3 cm in the East, North, and Up components with a fixing percentage of 96.8%.

Odolinski and Teunissen [27] comprehensively assessed the performance achievable by the u-blox zed-f9p by comparing the Best Integer Equivariant (BIE) and the Integer Least Square (ILS) estimators.

Furthermore, Nie et al. [28] proposed a new method that was revealed to be more suitable for mass-market applications with low-cost GNSS devices. The results of stationary and automotive experiments conducted with a low-cost single-frequency receiver showed that the proposed method can quickly reach half-meter accuracy in the horizontal at a much faster convergence speed than the conventional double-frequency PPP.

Robustelli et al. [29,30] obtained interesting results in terms of the time needed to resolve the phase ambiguity at integer values along with good accuracy and precision in a static test using the u-blox Point Perfect commercial PPP-RTK data correction service. Although there is an extensive literature on PPP-RTK theoretical approaches and implementation methods, as described in [31], the literature on the design of IoT location devices based on GNSS-sourced signals remains limited, as do case studies involving real-world testing. The development of engineered devices has room for improvement; hence, in this work we report the design and implementation of an Internet of Things (IoT) GNSS device developed with low-cost hardware that leverages a commercial PPP-RTK data correction service targeting horizontal and vertical decimetre level accuracies in static and kinematic tests. The novelty of this contribution mainly resides in (i) the sub-decimetre level of accuracy obtained with low-cost equipment, and (ii) the real-world kinematic testing of a low-cost commercial augmentation service exploiting a cutting-edge technique such as PPP-RTK. Alongside the accuracy target, other requisites include the minimum value of solution availability, the provision of connection ports for interfacing between the device and IoT network, and competitive pricing compared to low-cost receivers currently available on the market. Throughout this paper, the positioning accuracy is evaluated by comparing the positions obtained using the IoT device to reference positions calculated with a pair of geodetic-grade receivers. The remainder of this manuscript is organised as follows: the current “Introduction” section provides the motivation of the work and its context, along with a brief overview of the literature related to PPP-RTK. The “IoT Location Device Design and Implementation” section outlines the methodology followed in developing the IoT low-cost GNSS-based positioning device. The “Experimental Setup” section presents the experimental setup, detailing the leveraged hardware, software, and positioning techniques. The “Results” section explains the obtained results. The “Discussion” section discusses the results. Lastly, the “Conclusions” section draws conclusions and offers a brief outlook on future work.

## 2. IoT Location Device Design and Implementation

This section describes the design and implementation details of the IoT location device. The device was designed to be mounted on rovers and agricultural tractors, including autonomous ones. For this reason, in addition to collecting precise data, the device must be able to provide these data both via a serial connection for local use by the machinery, as well as on an IoT network to guarantee the interoperability of autonomous machinery and remote monitoring. The functional requirements listed in Table 1 formally specify the main features that the IoT location device has to provide.

There is a strong analogy between a single Thing in an IoT system and an embedded system [32]. Because the IoT location device can be considered as an embedded real-time software system, a component-based development process with separation of concerns [33] has been adopted. Moreover, to organise the development process in a manner that enforces separation of concerns in the design space, the concept of *“design view”* has been followed. The *“International Standard ISO/IEC/IEEE 42010 Systems and software engineering—Architecture description [34]”* stipulates that the *“Architectural description of the system is organised into one or more constituents called views,”* where a view is a partial representation of a system from a particular viewpoint which is the expression of some stakeholders’ concerns. During the construction of a development approach, if we ratify that each view is the expression of a single concern then these views become effective means to enforce separation of concerns in the specification of the software system. The development process that we followed aimed to implement the physical embedding, systems integration, and network connectivity as well as the software and application implementation [35]. In the following, the architectural design views of the device in compliance with the guidelines suggested by the 42010 international standard [34] are described. For each view, we indicate the adopted separation of concerns as indicated by [33].

### 2.1. Hardware and Deployment Views

**Figure 1:**
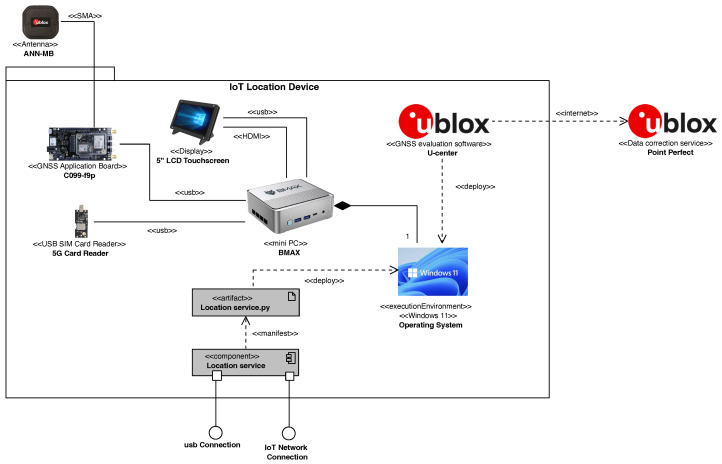
Combination of Hardware and Deployment views of the IoT location device.

The UML deployment diagram in Figure 1 shows a combination of hardware and deployment views of the IoT location device. The hardware view indicates the typologies of hardware components selected to design the device and their connections. As the figure shows, the IoT location device is centered purely on a physical node, which is a BMAX <<mini PC>> equipped with a Windows 11 Operating System acting as <<execution environment>>.

The mini-PC is physically connected through a USB cable to a C099-f9p <<GNSS Application board>> component, which in turn is connected to a low-cost GNSS ANN-MB <<antenna>> by means of a SubMiniature type A (<<SMA>>) cable. The <<GNSS Application board>> is responsible for receiving GNSS signals, constructing the GNSS observables, and finally calculating the positioning solutions. The C099-f9p <<GNSS Application board>> mounts the u-blox low-cost GNSS ZED-F9P GNSS module, which has 184 channels, to track the following signals:

1. GPS Coarse/Acquisition (C/A) code on L1 band and modernised L2C signal on L2 band;
2. GLONASS open access signals L1OF and L2OF on L1 and L2 bands, respectively;
3. Galileo E1B and E1C code for Open Service (OS), Safety of Life (SoL), and Commercial Service (CS) on E1 band and E5b signal on E5 band;
4. B1I and B2I Beidou signals;
5. QZSS L1C/A, L1S, and L2C signals;
6. L1 C/A SBAS.

The mini-PC hosts the <<GNSS evaluation software >> by u-blox (u-center ver. 21.09), utilizing the high-precision GNSS augmentation service (<<data correction service>>) called “Point Perfect”. This software acts as an interface with the GNSS receiver, facilitating the reception of corrections through either IP or L-band. In the present case, the IP network receives two types of SPARTN 2.0 format messages: satellite clock corrections every 5 s, and satellite orbits, bias, and atmosphere corrections every 30 s. The data correction service delivers corrections in ITRF2014 (the current epoch); hence, to compare the ground truth and PPP-RTK solutions, a transformation is applied to consider the relationship of the ETRS89 (in its realization ETRF2000) at epoch 2008.0 with the International Terrestrial Reference System (ITRS).

Furthermore, to allow interaction with the user, the BMAX is connected to an LCD Touchscreen <<display>> through a <<USB>> and an <<HDMI>> cable. We equipped the device with a <<USB SIM Card Reader>> to guarantee a 5G internet connection. Because the device should additionally provide connection ports allowing devices connected to the IoT network to gather location data in real time, we designed and implemented Location service.py in Python to manifest the Location service component.

**Figure 2:**
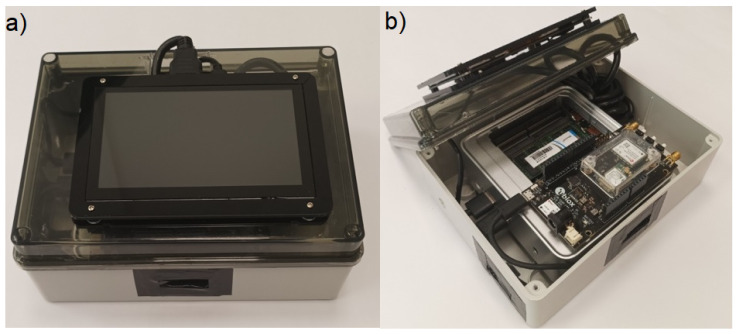
(**a**) Polycarbonate box with 5 inch screen for interfacing with the IoT system and (**b**) IoT Location Device Packaging (overall measurements: 19.5 cm × 15 cm × 9.5 cm).

To ensure that devices connected to an IoT network such as a 5G network can gather location data in real time, we designed and implemented the Location service.py in Python to manifest the Location service component responsible for providing the IoT Network Connection interface. Though not explicitly required, the Location service was equipped with a USB Connection interface through which other devices, such as autonomous farming machinery or rovers, can be directly connected. Details on the design and implementation of this component are described below. Figure 2 shows the packaging of the IoT location device. Panel (a) of Figure 2 shows the IoT device enclosed in its IP67 polycarbonate box; a 5 inch touchscreen allows for interaction by the user. Panel (b) of Figure 2 shows the internals of the box with all the hardware components connected.

From the cost perspective, the market value of the IoT location device is less than USD 550. At this date, to best of our knowledge, it can be considered low-cost compared to other devices able to achieve similar positioning performance.

### 2.2. Component View

**Figure 3:**
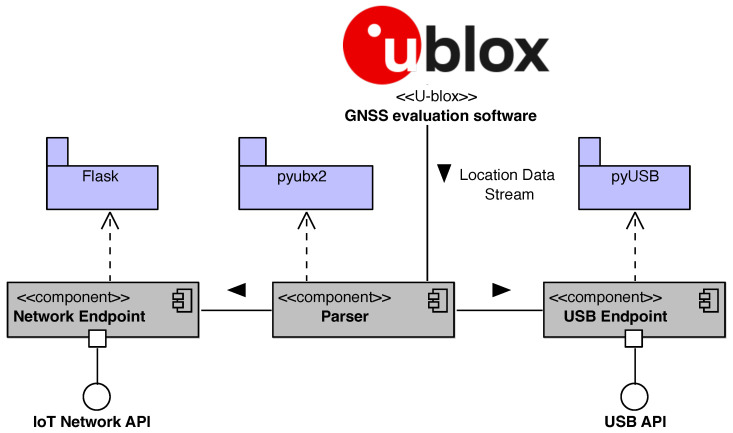
Component view of the Location service.

This paragraph describes the design and implementation of the Location service reported in Figure 1. Figure 3 shows the UML component diagram describing the internal architecture of the Location service in terms of its components, connectors, and third party libraries.

The component diagram’s main purpose is to show the structural relationships between the components of a system. Components are considered as autonomous encapsulated units within a system or subsystem that provide one or more interfaces. Components are larger design units that represent things that are typically implemented using *replaceable* modules. In component-based development (CBD), component diagrams offer architects a natural format to model a solution that can be implemented by selecting either off-the-shelf components or developing them from the scratch. The interfaces provided by each component represent the formal contract of services that the component provides to its consumers/clients. The interface “lollipop” symbols with a complete circle at the end represent an interface that the component provides. These symbols represent the interfaces through which a component produces information used by other components.

As shown in Figure 3, the component calculates the positioning solutions by exploiting the <<u-blox>> GNSS evaluation software for receiving data from satellites and PPP-RTK corrections via Internet Protocol (IP). The <<u-blox>> software stores the data in a u-blox proprietary file (UBX format). In addition, a python parser leveraging the pyubx2 (https://github.com/semuconsulting/pyubx2 (accessed on 10 October 2023 ) library, which provides a parser API for the UBX protocol (proprietary binary protocol implemented on u-blox GNSS/GPS receiver modules), has been developed and implemented; pyubx2 is capable of parsing NMEA 0183 and RTCM3 GNSS messages as well. At run-time, the component parses the Location data stream produced by the GNSS evaluation software, then sends the parsed data to the Network and USB Endpoint components. The Network Endpoint component is responsible for exposing the IoT Network API, which is actually a RESTful API that provides the get methods to connect the device over an the internet network. To implement this component, the Flask (https://flask.palletsprojects.com/en/3.0.x/ (accessed on 23 October 2023 )) library has been imported.

Similarly, the USB Endpoint component, implemented using the pyUSB (https://github.com/pyusb/pyusb (accessed on 9 November 2023 ) library, provides the USB API that can be used to export the parsed positioning data to other devices through a USB connection.

### 2.3. Data View

**Figure 4:**
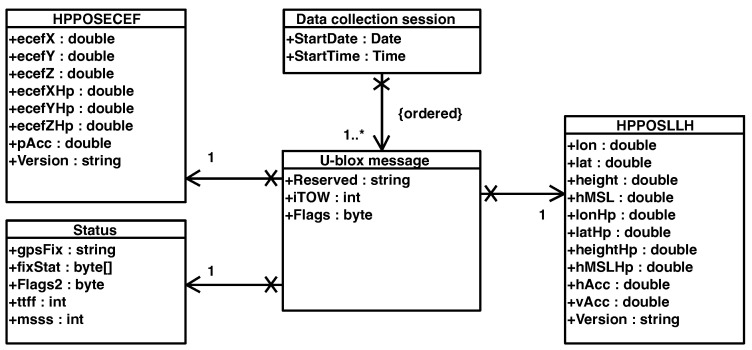
Data view.

The complete description of the <<u-blox>> protocol (https://content.u-blox.com/sites/default/files/documents/u-blox-F9-HPG-1.32_InterfaceDescription_UBX-22008968.pdf (accessed on 23 September 2023 ) defines all the messages that the user can activate on demand. The document describes the interface of the u-blox F9 high-precision GNSS receiver. The interface consists of different protocols, such as NMEA, UBX, RTCM, and SPARTN (Secure Position Augmentation for Real-Time Navigation) [36]. The latter is used to supply the GNSS receiver with real-time correction data. The SPARTN 2.0 support is implemented according to its Interface Control Document (https://www.spartnformat.org/wp-content/uploads/220221_SPARTN_v2.0.2.pdf (accessed on 7 September 2023). On the other hand, the UBX protocol is required to output high precision solutions. The UML class diagram in Figure 4 shows the data view of the signals parsed by the Location service component. As indicated in the figure, the parser generates a Data collection session with a starting date and time.

A Data collection session contains an ordered list of parsed u-blox messages. Specifically, three required sub-messages inside the UBX protocol are activated: HPPOSECEF, HPPOSLLH, and Status. The first two contain the high-precision positioning solutions in geocentric Cartesian (ECEF) and ellipsoidal coordinates, whereas the latter holds the information about the corresponding solution status, e.g., single point, code differential, PPP-RTK Float, and PPP-RTK Fixed. The snippet of code shown in Listing 1 reports an example of code written in Python for: (i) parsing every 0.01 sec the UBX messages coming from a serial connection; (ii) packaging the parsed data in JSON messages; and (iii) sending the JSON messages to a service that we implemented for storing the data.

**Listing 1.** Snippet of Python code for parsing UBX messages and sending the parsed data (in JSON format) to a web service for data storing.

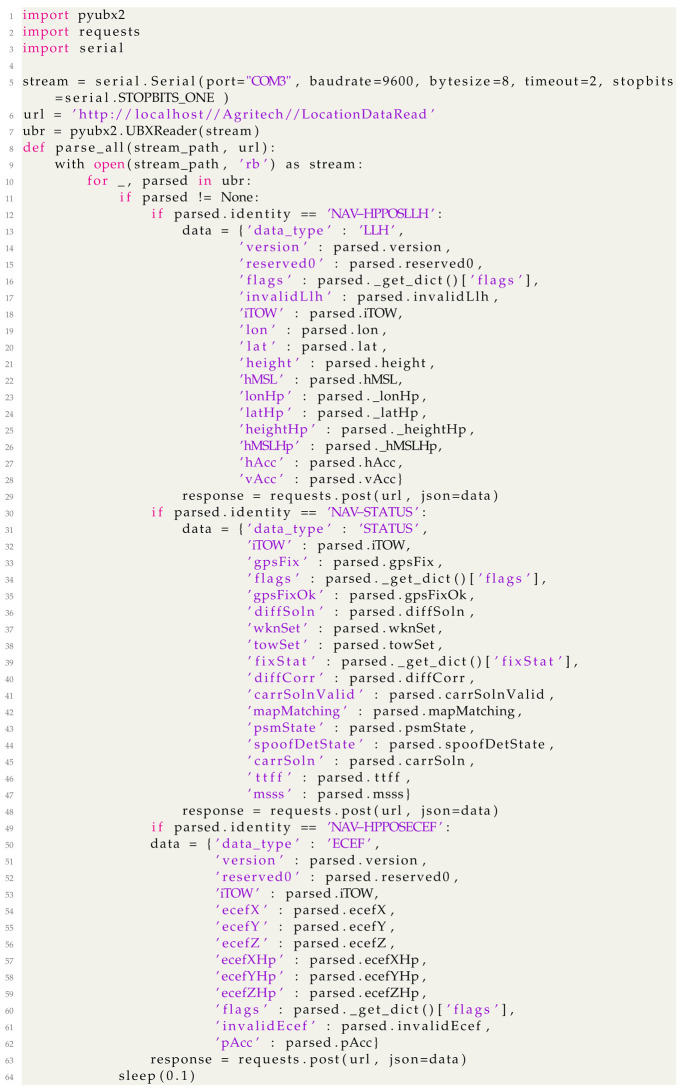



## 3. Experimental Setup

**Figure 5:**
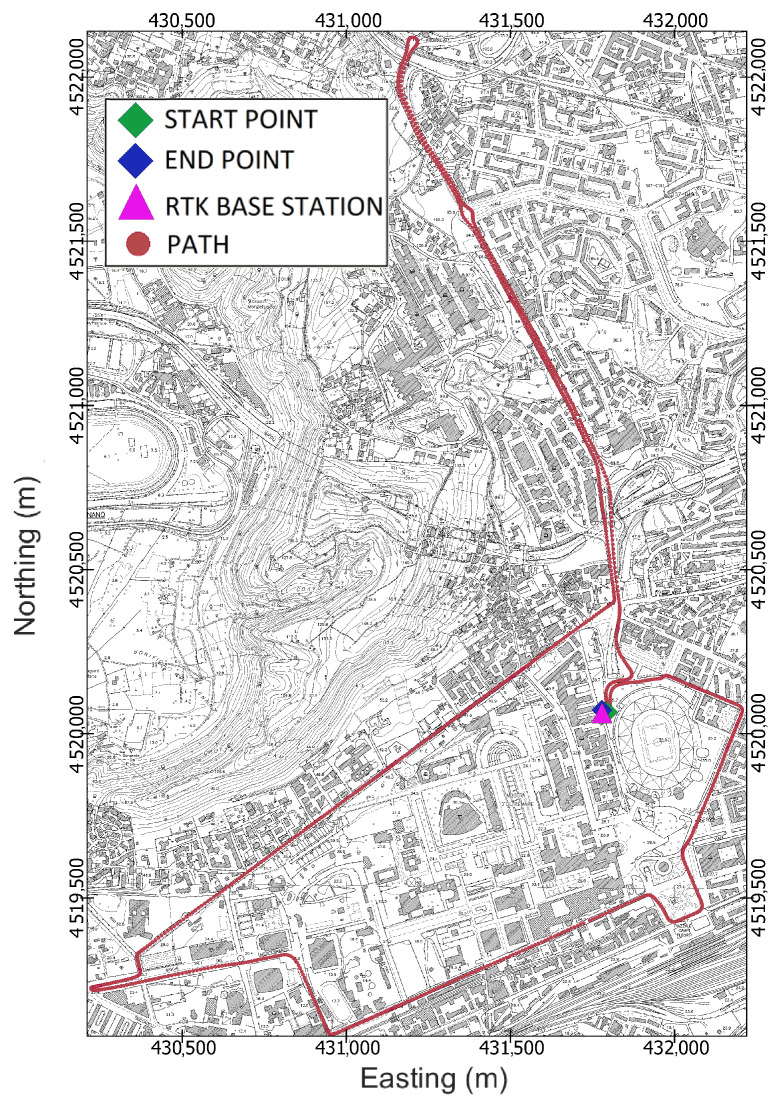
Cartographic map of Fuorigrotta neighbourhood in Naples, Italy showing identification of the test track (red circles). The figure shows the start point (green marker), end point (blue marker), and location of the GNSS base station (magenta marker).

The goal of the experimentation was to evaluate the accuracy and precision of the positioning solutions delivered by the IoT location device against a ground truth. To achieve this goal, the test path shown in Figure 5 was designed so as to be strongly heterogeneous from the perspectives of both satellite visibility and the presence of multiple paths. The path comprehends parts with almost ideal environmental conditions, e.g., open sky, along with other parts that can be considered as urban canyons, where trees and high buildings block the sky and a high multipath effect can be considered likely. The track length is 7.9 km, and about 20 min were necessary to complete it entirely.

**Figure 6:**
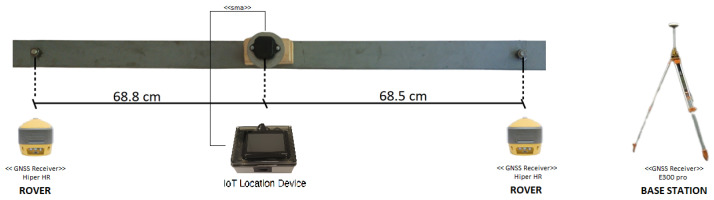
Experimental setup, including the aluminium bar used for mounting the two geodetic-grade GNSS receivers (at the ends of the bar) and the low-cost GNSS antenna feeding the IoT GNSS receiver. The right part of the figure shows the GNSS base station mounted on a tripod.

**Figure 7:**
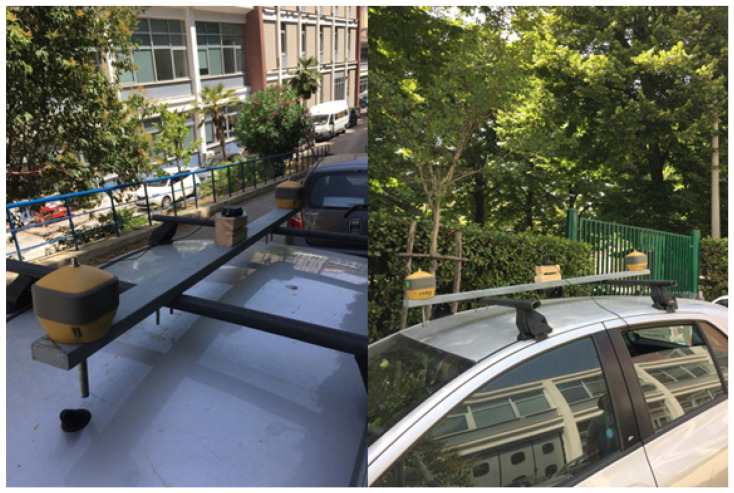
Images depicting the bar mounted on the test car. The left image displays a frontal view, while right image displays a side view. It is worth noting that a wood base of a certain thickness was employed to align the phase centres of the three antennas.

Afterwards, to obtain the ground truth, a pair of geodetic-grade Topcon Hiper HR GNSS receivers serving as rovers were mounted at the end of an aluminium bar designed for the purpose and placed above the test vehicle, as shown in Figure 6. Moreover, the experimental setup was completed by placing a base station on a point of known coordinates. The location of the base station, depicted in Figure 5 by a magenta triangle, was chosen to minimise the Post-Processing Kinematic (PPK) baseline. Figure 7 reports two images of the car roof equipped with the described instrumentation. The GNSS antenna setup was designed to allow the epoch-by-epoch computation of the true installation point of the low-cost antenna; hence, the positioning errors of the solutions provided by the IoT location device were calculated with respect to this reference.

Lastly, an urban kinematic test was conducted along the designed path on 19 July 2023 in Naples, Italy.

The GNSS was configured to a sampling rate of 1 Hz. Regarding the velocity, it is worth noting that the test was conducted in an urban scenario where vehicular traffic and traffic lights resulted in an extremely variable velocity over the track, ranging from 0 to 35 km/h.

## 4. Results

**Figure 8:**
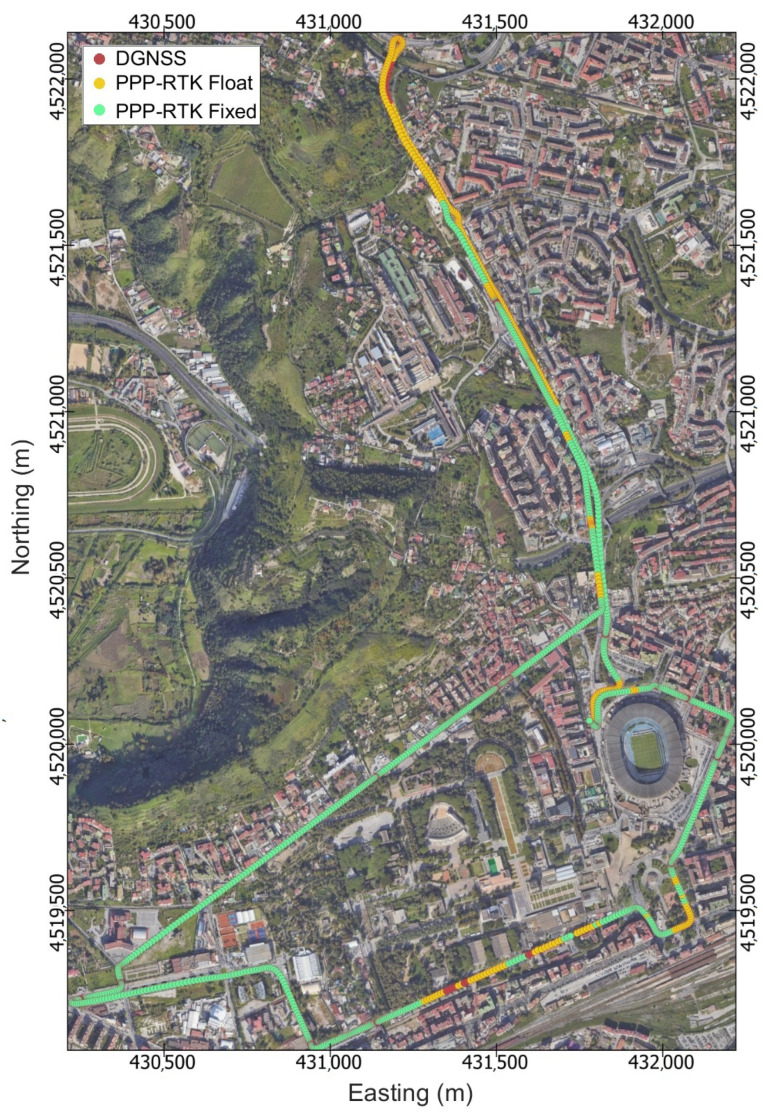
IoT location device positioning solutions for the entire kinematic test. The markers colours have been chosen for better visualisation. Green markers refer to PPP-RTK fixed, yellow makers to PPP-RTK float, and red markers to code differential (DGNSS).

Figure 8 shows the map reporting the position solutions on a satellite view background. The marker colours have been chosen for better visualisation; the green circles refer to PPP-RTK fixed solutions, the yellow circles to PPP-RTK float solutions, and the red markers to DGNSS solutions. The IoT Location device computed 1378 positioning solutions, of which 79.5% had phase ambiguity resolved at integer values, 19.2% had phase ambiguity resolved at float values, and 1.3% had code differential solutions. The float and DGNSS solutions were mainly grouped in two areas around the most northerly and south-easterly parts.

**Figure 9:**
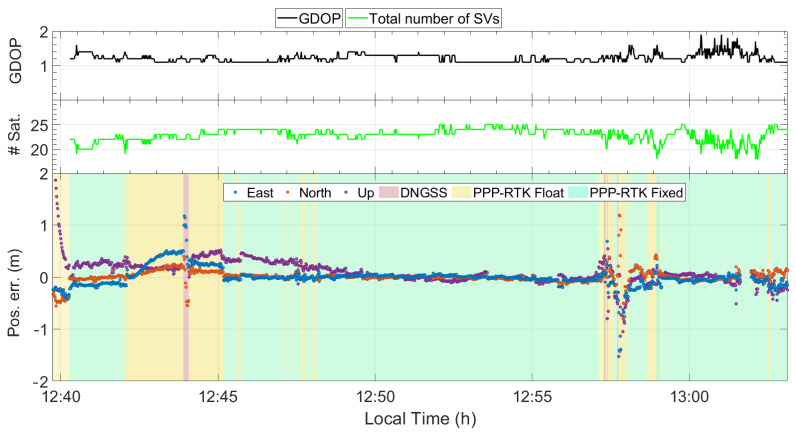
Geometric Dilution of Precision (GDOP), number of satellites, and time series of East, North, and Up coordinate component errors; the green, yellow, and red background colours refer to PPP-RTK fixed, PPP-RTK float, and code differential (DGNSS) solutions, respectively.

Figure 9 illustrates the time series detailing errors in the East, North, and Up coordinate components, and additionally includes the associated number of satellites and the Geometric Dilution of Precision (GDOP). The errors in the East, North, and Up directions are represented by blue, red, and yellow markers, respectively, while the parts with green, yellow, and red background colors correspond to PPP-RTK fixed, PPP-RTK float, and DGNSS solutions, respectively.

Considering the bottom part of Figure 9, it can be seen that the float solutions are mainly grouped within the time intervals from 12:43 to 12:45 and from 12:57 to 12:58; these were identified as the more unfavourable for the GNSS receiver due to the presence of reflective features resulting in a high multipath effect. Specifically, the leftmost yellow region corresponds to areas with high buildings on the left side and trees on the right side, whereas the rightmost yellow region corresponds to a scenario that can be properly assimilated to an urban canyon, as the street is surrounded by tall trees and buildings on both sides. Conversely, the figure reports extended parts (the green background) where the GNSS receiver was able to resolve and maintain the ambiguity resolution at integer values. These parts mostly correspond to favourable scenarios, except for the rightmost region, which corresponds to an urban scene due to the presence of the “Diego Armando Maradona” football stadium and the medium-size buildings on the other side.

**Figure 10:**
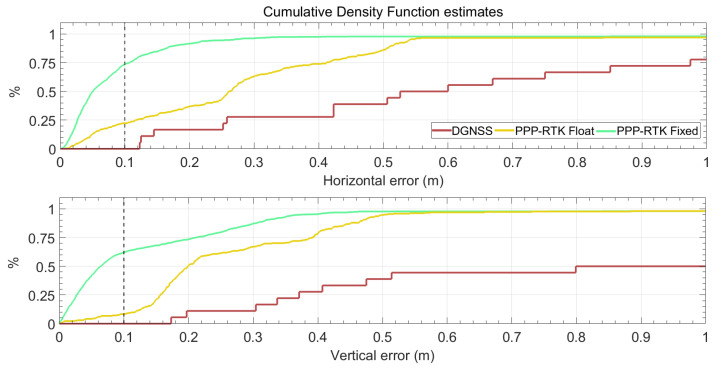
Cumulative density function estimates of positioning errors for the entire track. The green and yellow lines refer to PPP-RTK fixed and float solutions, respectively, while the red lines refer to code differential (DGNSS) solutions. The **top panel** and **bottom panel** refers to the horizontal and absolute values of the vertical error, respectively.

Figure 10 reports the cumulative density function estimates of the horizontal and vertical positioning errors in the top and bottom panels, respectively. The green, yellow, and red lines refer to PPP-RTK fixed, PPP-RTK float, and DGNSS solutions, respectively. The top panel of Figure 10 highlights that when the phase ambiguity is resolved at integer values, about 75% of the solutions have a horizontal error of less than 0.1 m and 96% show a horizontal error below 0.3 m. As expected, the performance worsens when the solution is of the float type; only 22% of the float solutions show a horizontal error below 0.1 m, whereas 63% show a horizontal error below 0.3 m. The bottom panel of Figure 10 indicates that the solution accuracy is worse when compared to horizontal one, as when the phase ambiguity is resolved at integer values only 62% of the solutions have an absolute value of the vertical error of less than 0.1 m and 87% show an error below 0.3 m. Furthermore, when the solution is of the float type, 8% of solutions show an error below 0.1 m, whereas 67% show an error below 0.3 m.

**Figure 11:**
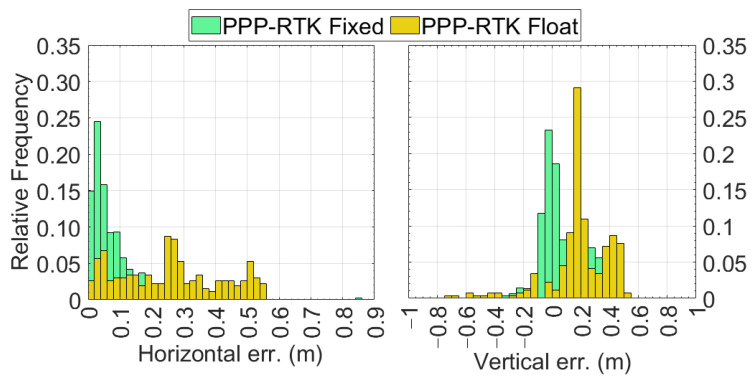
Relative frequency histograms of the horizontal (**left panel**) and vertical (**right panel**) errors. The green bars correspond to PPP-RTK fixed solutions, while the yellow bars refer to PPP-RTK float solutions.

Figure 11 reports the relative frequency histograms of the horizontal (left panel) and vertical (right panel) errors. It can be observed that when the ambiguity is fixed at integer values, the horizontal error is under 0.02 m in 16% of the epochs, whereas for 26% of the epochs the error is between 0.02 and 0.04 m. Moving on to the solutions with float-type ambiguities, it can be seen that the yellow bars corresponding to an error up to 0.20 m are always lower than the green ones. This highlights that the percentage of epochs with errors lower than 0.10 m is lower when the ambiguity is of float type. The situation changes completely as the error increases; starting from 0.20 m, the yellow boxes are always higher than the corresponding green ones. The right panel of Figure 11 reports the relative frequency histograms of the vertical errors. As expected, this panel includes also negative numbers. In this case, the distribution of vertical errors of the epochs for which the ambiguity is resolved at integer values are centred around zero, while they are shifted of about 0.20 m in the case of float type solutions.

**Figure 12:**
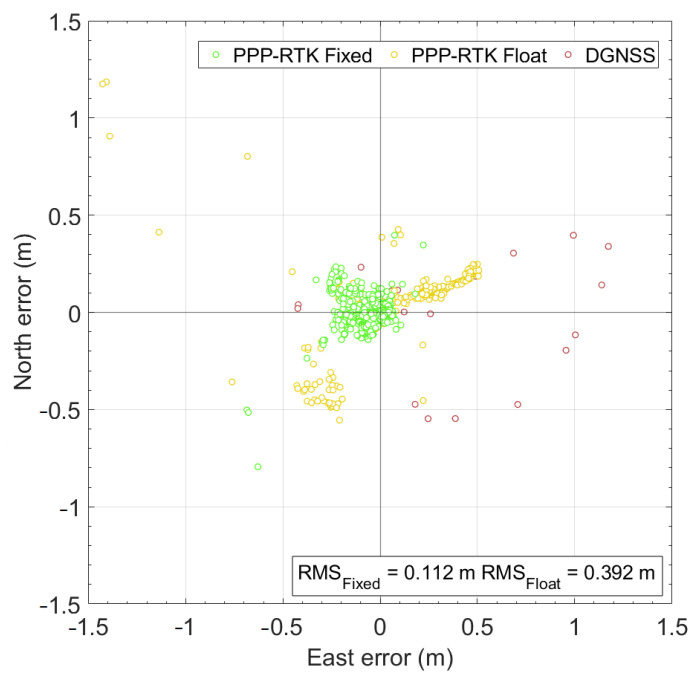
Scatter plot of the horizontal coordinate component errors for the entire path. The red, yellow, and green markers refer to DGNSS, PPP-RTK float, and PPP-RTK fixed solutions, respectively.

Lastly, Figure 12 reports the scatter plot of the horizontal positioning errors. It is worth noting that PPP-RTK solutions with ambiguity resolved at integer values (green circles) achieve an RMS of 0.112 m, whereas the same is about three times greater (0.392 m) when the PPP-RTK solutions are of float type. As depicted in the figure, the RMS calculated for all the solutions is 0.222 m.

Table 2 reports the positioning metrics relative to the accuracy and precision obtained during the vehicle-born experiment, along with the number and percentage of solutions of different types. Regarding the horizontal positioning, the fixed solutions show a mean error of 0.077 m with a σ of 0.080 m. Considering the float solutions, the mean error degrades, as expected; indeed, the mean horizontal error of the float solutions is equal to 0.310 m, with a σ of 0.240 m. By analysing these values, it can be noticed that the mean errors of the fixed solutions are four to five times less than the float solutions in terms of accuracy. When looking at the precision, as quantified by σ , the fixed solutions are three times lower. Regarding the vertical positioning, the accuracy is maintained under the target threshold, e.g., 0.1 m, as the mean vertical error for fixed solutions is equal to 0.052 m, whereas for the float solutions it reaches a value of 0.272 m. The vertical precision, however, is heavily degraded, as the σ values for fixed and float solutions are 0.163 and 0.471 m, respectively.

## 5. Discussion

In the previous sections, the description of the design, implementation, and kinematic experimentation of an IoT location device has been reported. This is devoted to verifying whether or not the system achieves the requirements specified in the “IoT Location Device Design and Implementation” section.

### 5.1. IoT Location Device Performance in Static Test (Rq ${\;}_{1}$ and Rq ${\;}_{2}$ )

According to the results obtained in [30], it can be stated that, in static conditions, the system can reach a horizontal accuracy (as measured by the RMS error) equal to 0.072 m for fixed solutions and 0.406 m for float solutions. The corresponding precision values, as measured by σ , are 0.089 and 0.656 m, respectively. Concerning the vertical accuracy, it can be stated that in static conditions the system can reach a vertical accuracy (as measured by the RMS error) equal to 0.204 m for fixed solutions and 0.877 m for float solutions. The corresponding precision values, as measured by σ , are 0.284 and 1.905 m, respectively. Considering that the system was able to deliver solutions with phase ambiguity resolved at integer values for about the 94% of usage time, Rq ${\;}_{1}$ and Rq ${\;}_{2}$ can be considered satisfied.

### 5.2. IoT Location Device Performance in Kinematic Test (Rq ${\;}_{3}$ and Rq ${\;}_{4}$ )

Moving to the vehicle-born test results (see “Results”), it has been discovered that in kinematic conditions the receiver can reach a horizontal accuracy (as measured by μ ) equal to 0.134, with a precision (measured by σ ) equal to 0.177 m and an RMS of 0.222 m. Considering only solutions with phase ambiguity resolved at integer value, the mean error, σ , and RMS are 0.077, 0.080, and 0.112 m, respectively. Conversely, considering only float solutions, the accuracy and precision metrics are 0.310, 0.240, and 0.392 m, respectively. For the vertical component, the IoT location device can reach an accuracy equal to 0.122 m in terms of the mean error. The corresponding precision as measured by σ is 0.408 m. Lastly, the RMS for the vertical component is equal to 0.427 m. When considering only fixed solutions, the values are 0.052, 0.163, and 0.171 m, respectively. Conversely, for float solutions the mean error, σ , and RMS are 0.272, 0.471, and 0.546 m, respectively. These results verify that targets Rq ${\;}_{3}$ and Rq ${\;}_{4}$ are achieved.

### 5.3. IoT Location Device Solution Availability (Rq ${\;}_{5}$ )

Along with the accuracy targets, a solution availability requirement was set. In this regard, according to the top panel of Figure 10, the IoT location device delivers solutions with a horizontal error less than 0.30 m for about 90% and 60% of the usage time for fixed and float solutions, respectively. Concerning the solution availability corresponding to the target vertical accuracy, as shown in the bottom panel of Figure 10, the developed IoT location device can reach the targets for about 87% and 65% of the usage time for fixed and float solutions, respectively.

### 5.4. IoT Location Device Connectivity (Rq ${\;}_{6}$ )

**Figure 13:**
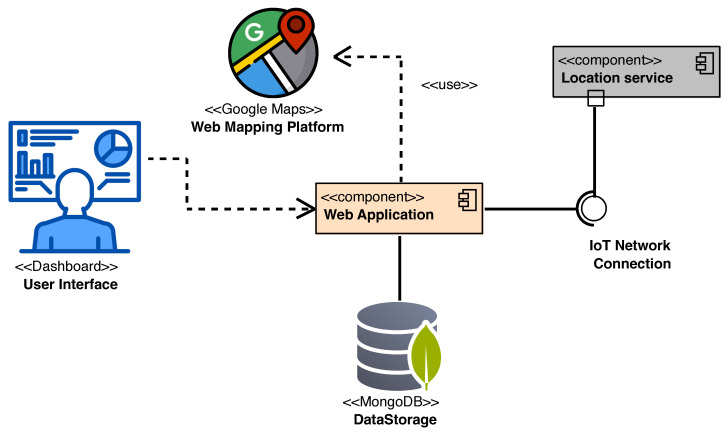
Screenshot of the developed web application.

The RESTful APIs provided by the Location service component were exploited to implement a web application, the architecture of which is depicted in the UML component diagram shown in Figure 13.

**Figure 14:**
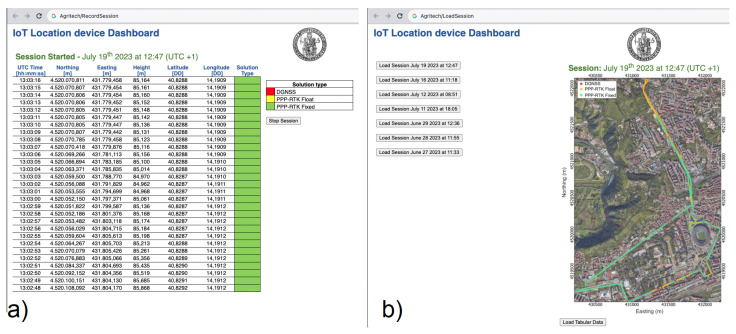
Panel (**a**) record session dashboard. Panel (**b**) load and view stored sessions dashboard.

As reported in the figure, the web application exploits the APIs provided by the Location service component to gather the positioning data and to provide them to a User Interface rendering one or more <<Dashboards>> such as the ones shown in Figure 14.

The web app mainly provides two dashboards. The one depicted in panel (a) of Figure 14 is used to record a session. During a recording session, the table shown in the panel is updated in real time with the epoch-by-epoch positioning data, reporting the UTC time, geographic and Cartesian coordinates, and corresponding solution status. At the end of the recording session, the positioning data are saved in the data storage implemented in <<MongoDB>>. Each stored session is named with the date and starting time of recording. The second dashboard provided by the web app, shown in panel (b) of Figure 14, permits the loading of stored sessions. When loaded, the data can be visualised in either a tabular view or a 2D map view. The latter view displays the recorded positions on a Google map satellite view, with each marker is coloured according to the solution status.

## 6. Conclusions

In conclusion, the successful achievement of certain and predefined accuracy levels and solution availability along with the implementation of connectivity features makes the developed IoT location device a potential solution to meet the need for a cost-effective positioning system for mass-market kinematic applications. As expected, the resolution of phase ambiguity at integer values emerges as a crucial factor contributing to the system’s effectiveness. Indeed, when the GNSS receiver is able to resolve the phase ambiguity at integer values, the positioning accuracies are always at the centimetre level. As shown in the“Discussion” section, this condition is maintained during 79.5% of the device’s usage time, while in 19.2% of the time the receiver delivers positioning solutions with decimetre-level accuracy. PPP-RTK corrections were not available at all in the remaining 1.3% of the time; hence, the DGNSS technique delivered positioning solutions at the metre level. Furthermore, the integration of the device into an IoT network was facilitated by its RESTful APIs, which were utilised to create a web application featuring a comprehensive dashboard displaying real-time positions and the device’s route on a Google map. Regarding cost-related aspects, the IoT location device proves to have low cost compared to other devices offering similar positioning performance. In our future work, we plan to extend field experimentation with the IoT location device in precision farming and smart agricultural applications. Moreover, there is room to improve the device’s cost-effectiveness by using alternative low-cost hardware solutions such as the Ardusimple RTK2B for the GNSS receiver side and the Raspberry Pi5 for the mini-PC side. Our next efforts in testing and optimising the device will schedule solution rates up to 10 Hz, allowing a solution to be delivered at each 28 cm for a tractor moving at 10 km/h. An additional research area concerns reducing both the size and power consumption of the prototype, as these two characteristics can be possible limitations when embedding devices in many application domains. The current prototype does not have such limitations related to integration in rovers and agricultural tractors, as the device is 19.5 cm × 15 cm × 9.5 cm in size and can be easily mounted on these types of machines. Similarly, the power consumption cannot be considered a limitation for the considered domain, as testing demonstrated the installation of the prototype in a car where it was fed by the 12 V furnished by the car’s battery. A further research direction is the integration of an IMU to improve the robustness of IoT location device solutions and implement bridging capabilities. Additional application fields to be explored in the future relate to the use of the IoT location device for landslide monitoring as well as its integration in autonomous rovers and farming robots. 

## Figures and Tables

**Table 1:** Functional requirements of the IoT location device.

Rq_ID_	Description
Rq ${\;}_{1}$	the device has to deliver positioning solutions with a horizontal target accuracy and precision at a sub-decimetre level in static conditions
Rq ${\;}_{2}$	the device has to deliver positioning solutions with a vertical target accuracy and precision at a decimetre level in static conditions
Rq ${\;}_{3}$	the device has to deliver positioning solutions with a horizontal target accuracy and precision at a decimetre level in kinematic conditions
Rq ${\;}_{4}$	the device has to deliver positioning solutions with a vertical target accuracy and precision at a decimetre level in kinematic conditions
Rq ${\;}_{5}$	the device has to reach an adequate level of solution availability (90% of usage time) maintaining a solution accuracy up to 0.3 m
Rq ${\;}_{6}$	the device has to provide connection ports to be connected to an IoT network

**Table 2:** Summary table of the positioning performance of the IoT location device. Note that DGNSS statistics were not calculated due to the low number of results for this solution type.

			Horizontal (m)			Vertical (m)		
Solutions	# *sol*.	%*sol*.	*μ*	*σ*	*RMS*	*μ*	*σ*	*RMS*
DGNSS	18	1.3%	-	-	-	-	-	-
Float	265	19.2%	0.310	0.240	0.392	0.272	0.471	0.546
Fixed	1095	79.5%	0.077	0.080	0.112	0.052	0.163	0.171
All	1378	100%	0.134	0.177	0.222	0.122	0.408	0.427

## Data Availability

Some or all data, models, or code that support the findings of this study are available from the corresponding author upon reasonable request.

## Abbreviations

The following abbreviations are used in this manuscript:

API	Application Programming Interface
CORS	Continuously Operating Reference Station
DGNSS	Differential GNSS
ETRS	European Terrestrial Reference System
GDOP	Geometric Dilution of Precision
GNSS	Global Navigation Satellite System
IMU	Inertial Measurement Unit
IoT	Internet of Things
IP	Internet Protocol
ITRF	International Terrestrial Reference Frame
ITRS	International Terrestrial Reference System
MQTT	Message Queue Telemetry Transport
NMEA	National Marine Electronics Association
NRTK	Network Real-Time Kinematic
OSR	Observation State Representation
PPK	Post-Processing Kinematic
PPP	Precise Point Positioning
PPP-RTK	Precise Point Positioning–Real-Time Kinematic
RTCM Radio	Technical Commission for Maritime Services
RTK	Real-Time Kinematic
SPARTN	Secure Position Augmentation for Real-Time Navigation
SSR	State Space Representation
TC	Tightly Coupled
UML	Unified Modeling Language
USB	Universal Serial Bus
UTC	Universal Time Coordinated
